# Comparison of screening accuracy of the Patient Health Questionnaire-2 using two case-identification methods during pregnancy and postpartum

**DOI:** 10.1186/s12884-020-02891-2

**Published:** 2020-04-14

**Authors:** Valerie Slavin, Debra K. Creedy, Jenny Gamble

**Affiliations:** 1 Transforming Maternity Care Collaborative, School of Nursing & Midwifery, Griffith University, Logan Campus, University Drive, Meadowbrook, Qld 4131 Australia; 2 Women, Newborn & Children’s Services, Gold Coast University Hospital, 1 Hospital Boulevard, Southport, Qld 4215 Australia

**Keywords:** Patient Health Questionnaire (PHQ), Depression, Pregnancy, Postpartum, Case-identification, Screening tool, Screening accuracy, Outcome measurement instrument, Patient reported outcome measure (PROM), Core outcome set

## Abstract

**Background:**

Variation exists regarding perinatal depression screening. A two-step screening method has been recommended. According to a maternity-focused core outcome set developed by the International Consortium for Health Outcomes Measurement, women who score 3 or more on the PHQ-2 then complete the Edinburgh Postnatal Depression Scale (EPDS). Limited evidence exists regarding the screening accuracy of the PHQ-2 in childbearing women. An alternative case-identification method may be more sensitive for perinatal women. We aimed to [1] evaluate the screening accuracy of the PHQ-2 during the perinatal period using two case-identification methods, and [2] measure the variability of accuracy over four time-points during pregnancy and postpartum.

**Methods:**

A prospective, longitudinal cohort study was conducted with 309 consecutive women who completed the PHQ-2 and EPDS during pregnancy (booking, 36-weeks) and postpartum (6-, 26-weeks). EPDS was the reference standard using cut-off scores for ‘*at least probable minor depression*’ during pregnancy (≥ 13) and postpartum (≥ 10) and for ‘*probable major depression*’ during pregnancy (≥ 15) and postpartum (≥ 13). PHQ-2 was analysed using two methods: [1] scored (cut-points ≥ 2 and ≥ 3), [2] dichotomous yes/no (positive response to either question) against EPDS cut-points for at least probable minor and probable major depression. Receiver operating characteristic analyses determined accuracy.

**Results:**

Probable major depression: Over four timepoints PHQ-2 ≥ 3 revealed lowest sensitivity (36–79%) but highest specificity (94–98%). An alternative case-identification method revealed high sensitivity (93–100%), but lowest specificity (58–71%). Minor depression: PHQ-2 ≥ 3 revealed the lowest sensitivity (19–50%) but highest specificity (95–98%). An alternative case-identification method revealed the highest sensitivity (81–100%) and moderate specificity (60–74%).

**Conclusions:**

Recommended method of case-identification (PHQ-2 ≥ 3) missed an unacceptable number of women at-risk of depression. As a clinical decision-making tool, an alternative, dichotomous method maximized case-identification and is recommended. Further, the literature identified inconsistent reporting of the PHQ-2 and the alternative case-identification method hindering the ability to synthesise data. The future use and reporting of consistent question wording and response format will improve outcome reporting and synthesis. Further research in larger and diverse maternity populations is recommended.

## Background

In Australia, around one in ten women experience depression during pregnancy [1] and one in six during the year following birth [2]. Untreated maternal depression has been consistently associated with poorer outcomes for infants including impaired attachment and cognitive deficits, with effects still adversely impacting on children at age 16, especially boys [3]. If not addressed, perinatal depression can create intergenerational difficulties [4]. In extreme cases, women may attempt or complete suicide or infanticide [5, 6]. The high burden of perinatal depression demands effective strategies to prevent and improve symptoms. Significant variation in depression outcomes, measures, and case definitions in comparative effectiveness trials limits data synthesis [7], and contributes to significant research wastage in perinatal research [8].

To address such issues the Core Outcome Measures in Effectiveness Trials (COMET) [9] and the Core Outcomes in Women’s and Newborn health (CROWN) [10] Initiatives advocate a standardized research approach using core outcome sets. A core outcome set is an agreed set of outcomes that should be measured and reported, as a minimum, in all clinical trials of specific health or health care [11]. In 2016 the International Consortium for Health Outcomes Measurement (ICHOM) published a standard set of outcome measures to evaluate value in maternity care [12]. Standard sets are the same as core outcome sets but have a clear focus on clinical practice [13]. An ICHOM working party comprising two consumers and nineteen international experts convened to identify outcomes and measurement instruments for inclusion in their set [14]. Using a modified Delphi technique and consensus process, mental health was identified as an outcome important to women, and the Patient Health Questionnaire (PHQ-2) [15] and Edinburgh Postnatal Depression Scale (EPDS) [16] were identified as the most appropriate measures of symptoms of perinatal depression to be included in the set.

ICHOM [12, 14] recommends the two-item PHQ-2 as a case-identification method for all women, followed by the 10-item EPDS *only* for women who screen positive on the PHQ-2 (defined cut-point of 3 or more). The sensitivity and specificity of the PHQ-2 to identify childbearing women at risk of depressive symptoms, as defined by accepted cut-points on the EPDS, is under-researched. As part of a core outcome set designed for use in clinical practice, high sensitivity on the PHQ-2 is vital to ensure all at-risk women also receive the EPDS. While probable major depression is the focus of ICHOM’s recommendation, minor depressive symptoms are also linked to poor quality of life and are important to consider during clinical decision-making [17].

### Clinical recommendations for depression screening

Screening women for depression during the perinatal period may reduce depressive symptoms and prevalence [18] but there lacks international consensus regarding the best approach. In Australia, where the current study is conducted, a universal screening approach is recommended at least twice during pregnancy (early and late pregnancy), and at least twice in the first postpartum year [19]. While a universal screening approach is also recommended in the United States [20], and Canada [21], the United Kingdom (UK) recommend selective screening adjunct to clinical practice [7]. Like the ICHOM approach, UK clinicians ask two case identification questions at first contact during pregnancy and again during the early postpartum period with further assessment only for women who respond positively to either question.

### Relevant depression screening instruments

The EPDS is the most widely-used, evaluated and validated measure of depressive symptoms [22–24]. Most clinical guidelines, including Australia, recommend the use of the EPDS, either as a primary [19, 21] or second-step screen [7] to inform clinical decision making. In terms of case-identification methods, two questions originating from the PRIME-MD diagnostic interview [25], are asked:

1. *During the past month, have you been bothered by little interest or pleasure in doing things?*
2. *During the past month, have you been bothered by feeling down, depressed or hopeless?*

The questions can be asked using two formats that differ in terms of timing and response format. When framed to recall symptoms over the past month, using a ‘*yes’* or ‘*no’* response, the questions are known as the Whooley Questions [26]. In contrast, when framed to recall over the past 2 weeks, using a four-item response, the questions are known as the Patient Health Questionnaire-2 [15]. While ICHOM recommends the use of the PHQ-2 for case-identification, guidance in the UK recommends the Whooley Question approach. Evidence regarding the diagnostic accuracy of the two approaches come from two systematic reviews conducted in general populations. Findings from Manea et al., [27] showed the PHQ-2 to have moderate sensitivity (76%) at the recommended cut-point of three or more which improved to 91% at a lower cut point. Bosanquet and colleagues showed the Whooley Questions approach to have the highest sensitivity (95%) [28]. In terms of maternity-focused evidence, a review conducted by Mann and Gilbody [29] included both case-finding methods (PHQ-2 and Whooley Questions) to detect postpartum depression. With only one included paper, these authors concluded limited evidence in support of the case-finding questions to detect postpartum depression with more research needed. To inform the ongoing implementation of the ICHOM core outcome set in clinical practice, the current study aimed to: (1) evaluate the screening accuracy of the PHQ-2 during the perinatal period using two case-identification methods (reference standard: EPDS), and (2) measure the variability of accuracy over four time-points during pregnancy and postpartum.

## Methods

### Study design

The current study is part of a larger body of work. The MoMeNT study (Models Meeting Needs over Time) is a prospective, longitudinal, cohort study which aimed to (1) evaluate the effectiveness of midwife continuity of carer on perinatal mental health and mother infant bonding and (2) assess the feasibility of the ICHOM core outcome set in the Australian context and is fully described elsewhere [30]. Feasibility of the ICHOM core outcome set includes the psychometric evaluation of included measures. The current study is designed to address our feasibility aim and is reported in accordance with STARD (Standards for Reporting of Diagnostic) criteria [31, 32], see STARD Checklist [Additional File 1].

### Setting, participants and sample size

Participants were recruited from one publicly-funded tertiary referral hospital in south-east Queensland. Participants were required to be English-literate, aged 18 years or older, 27-weeks gestation or less and have access to email and mobile phone. Women under the current care of a psychiatrist were excluded. Data collection took place between August 2017 and January 2019. Sample size was calculated to evaluate the broad effect of model of maternity care on maternal health and wellbeing. To identify a mean difference (two-tail) with a 50% effect size, 5% estimated error and 95% power, 210 participants were required. To allow for 20% attrition 252 participants were needed.

### Defining perinatal mental health

Depression was operationalized using the definitions outlined by the American Psychiatric Association [33]. Depression describes the presence of sad, empty, irritable mood, accompanied by somatic and cognitive changes that significantly affect the individual’s capacity to function. Anhedonia describes markedly diminished interest or pleasure in almost all activities. Major depression is defined as the presence of five or more symptoms (depressed mood, anhedonia, weight change, sleep disturbance, psychomotor problems, lack of energy, excessive guilt, poor concentration, suicidal ideation) present during the same 2-week period and represent a change from previous functioning with at least one of the symptoms being either depressed mood or loss of interest or pleasure. Minor depression is defined as the presence of at least two depressive symptoms but does not meet criteria for major depression.

### Measures

Surveys included the PHQ-2 and EPDS. Woman-reported socio-demographic data known to influence maternal mental health were collected at baseline including age (years), parity (number of births after 20 weeks gestation), gestation (weeks), educational attainment (low: secondary school year 12 or less; high: completed apprenticeship, diploma or tertiary degree), relationship status (single or in a relationship), and weekly combined income (low: less than $1500: high: $1500 or more).

#### Patient Health Questionnaire (PHQ-2)

The PHQ-2 [15] screens for possible depression and anhedonia. The stem question asks, *“Over the last 2 weeks, how often have you been bothered by any of the following problems?”* The two items are, *“little interest or pleasure in doing things”* and *“feeling down, depressed, or hopeless”.* For each item, the response options are “*not at all*” (0), “*several days*” (1), “*more than half the days*” (2), and “*nearly every day*” (3). PHQ-2 scores range from 0 to 6. Higher score represents greater depressive symptoms.

#### The Edinburgh Postnatal Depression Scale (EPDS)

The 10-item EPDS is a self-report measure to screen women for symptoms of depression during pregnancy [34] and postpartum [16]. Questions are framed, ‘*In the past 7 days …*. ’ with a frequency-based response scored on a four-point Likert scale, scored from 0 to 3. Recommended cut-off scores for ‘*at least probable minor depression*’ during pregnancy (≥ 13) and postpartum (≥ 10) and for ‘*probable major depression*’ during pregnancy (≥ 15) and postpartum (≥ 13) were used [35]. EPDS question items and screening accuracy at several cut-point as reported by NICE [7] are presented [Additional file 2].

### Procedures

Consecutive, eligible women attending antenatal care with a midwife were approached about the study. Women who provided written informed consent were sent a survey link by email and text message. Women who failed to respond were sent two friendly reminders and one telephone follow-up call, 2 - 3 days apart. Follow-up surveys were sent at 36-weeks, and 6- and 26-weeks postpartum. Women who failed to respond to two consecutive surveys were deemed lost to follow-up. All surveys provided information and contact numbers for national support groups. Participants were also offered the opportunity to contact the project manager to discuss any negative feelings. Survey completion occurred outside of clinical care where universal screening for depression is attended. Women who screened positive were not followed up but helpline information was provided in each survey. Ethical approval was granted from relevant Hospital and Health Service (HREC/17/QGC/127) and University Human Research Ethics Committees (GU Ref No: 2017/625).

### Approach to analysis

Using SPSS version 25 [36] the PHQ-2 was analysed using two methods: (a) total score at two cut-points (≥ 2 and ≥ 3) and (b) dichotomous categorical variable (positive response to *either or both* questions). Consistent with the work of others [37, 38], the EPDS was the reference standard. EPDS total score was transformed to a binary variable to indicate *at least probable minor depression* using the cut-off score of ≥ 13 = positive during pregnancy, and ≥ 10 = positive postpartum. Cut-off scores of ≥ 15 and ≥ 13 were used to denote *probable major depression* during pregnancy and postpartum respectively [35]. Group differences in mental health during pregnancy and postpartum and for non-completing women at 26-weeks, were assessed using chi-square. Effect size was interpreted using Cohen’s criteria [39]. Missing data were managed using listwise deletion for computing total scale scores and Cronbach’s α and pairwise deletion for all other analyses. Prevalence of depression risk according to the EPDS and positive PHQ-2 responses are presented as frequencies and percentages with 95% confidence intervals using Clopper Pearson Exact Tests for binary probability [40]. Change in proportion of participants who screened positive on the EPDS and PHQ-2 across the four time-points (variability of accuracy) were assessed using Cochran’s Q Test. Significance values were adjusted by the Bonferroni correction for multiple tests. Significance was *p =* <.05. For brevity the term ‘**minor depression**’ is used to denote ‘*at least probable minor depression*’ and ‘**major depression**’ is used to denote ‘*probable major depression*’.

#### Internal consistency

The internal consistency of the EPDS and PHQ-2 were assessed at all four time-points. Cronbach’s alpha coefficients (α) exceeding 0.7 were considered acceptable [41]. For scales with few items the mean inter-item correlation (MIC) is more accurate and reported for the PHQ-2. An ideal range was considered 0.2–0.4 [42].

#### Criterion validity.

Screening performance of the PHQ-2, defined as ‘area under the curve’ (AUC), sensitivity, specificity, positive predictive value (PPV), negative predictive value (NPV), positive likelihood ratio (PLR) and negative likelihood ratio (NLR), were assessed against the EPDS total score cut-points using ROC (Receiver Operating Characteristic) analysis [43] at all four time-points. The standard error for the area was set as non-parametric with a 95% confidence interval. Area under the curve (AUC) was interpreted according to the criteria by Tape [44] as: AUC = 0.60–0.70 = poor, 0.70–0.80 = fair, 0.80–0.90 = good, 0.90–1.0 = excellent. For the purposes of informing the ICHOM core outcome set and achieve a maximum likelihood that all ‘*at risk*’ women would be administered the EPDS, the optimal cut-off value on the PHQ-2 was set at 100% sensitivity.

## Results

### Sample characteristics

**Fig. 1:**
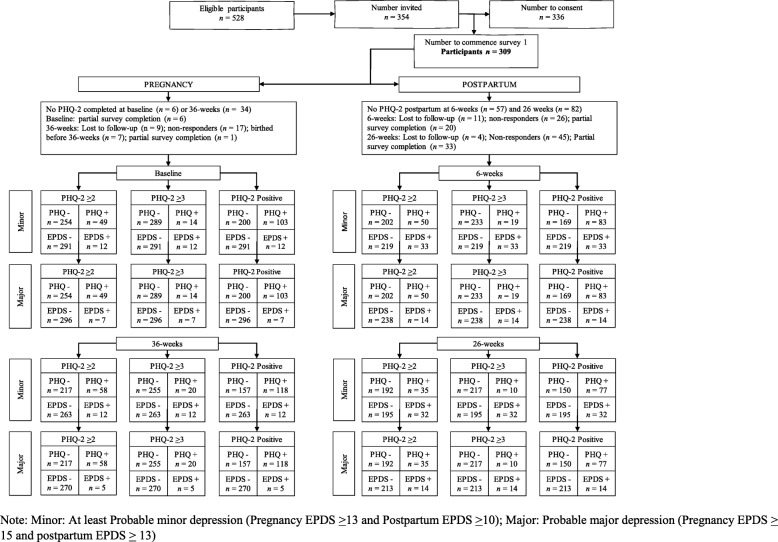
Standards for Reporting of Diagnostic Accuracy (STARD) flowchart with overview of participant selection. Note: Minor: At least Probable minor depression (Pregnancy EPDS ≥ 13 and Postpartum EPDS ≥ 10); Major: Probable major depression (Pregnancy EPDS ≥ 15 and postpartum EPDS ≥ 13)

A STARD diagram presents data pertaining to recruitment, attrition and cross-tabulation of index test/reference standard (Fig. 1*)*. The first survey was commenced by 309 pregnant women between 10 and 27-weeks gestation (*M* = 19.7, *SD* = 3.7). Table 1 presents group differences for women exceeding the EPDS cut-point for *at least probable minor depression* during pregnancy and postpartum and those below the cut-point. In early pregnancy, women who exceeded the cut-point (minor depression) were more likely to report a past history of mental health disorder (18.6% vs 1.5%, medium effect), report current cigarette use (37.5% vs 3.1%, medium effect) and lower income (7.8% vs 1.9%, small effect), compared to their non-depressed counterparts. At 26-weeks postpartum, only a history of mental health disorder remained significant (33.3% vs 10.8%, medium effect). There were no significant differences between women who remained in the study and those who did not (Table 1).

**Table 1:** Group differences for women with depression^±^ during pregnancy and postpartum, and for responders versus non-responders

	Pregnancy Baseline^**±**^				Postpartum 26-weeks^**±**^				Postpartum 26-weeks			
	***Depressed***	***Not depressed***	***P***^***#***^	***phi***	***Depressed***	***Not depressed***	***p***^***#***^	***phi***	Responders *	***Non-responders***	***p***^***#***^	***phi***
Age			.83	.03			.99	.02			.48	.05
≥ 35	3 (5.4)	53 (94.6)			7 (15.2)	39 (84.8)			46 (82.1)	10 (17.9)		
< 35	9 (3.6)	238 (96.4)			25 (13.8)	156 (86.2)			194 (76.7)	59 (23.3)		
Relationship status			.33	.09			.96	.03				
In a relationship	10 (3.5)	275 (96.5)			31 (14.4)	185 (85.6)			229 (79.0)	61 (21.0)	.06	.12
Single	2 (11.1)	16 (88.9)			1 (9.1)	10 (90.9)			11 (57.9)	8 (42.1)		
Education			.26	.06			.93	.02			.18	.09
Low	2 (2.1)	93 (97.9)			10 (15.2)	56 (84.8)			71 (72.4)	27 (27.6)		
High	10 (4.8)	198 (95.2)			22 (13.7)	139 (86.3)			169 (80.1)	42 (19.9)		
Income			**.04**	**.14**			.80	−.03			.19	.09
High	3 (1.9)	155 (98.1)			17 (13.6)	108 (86.4)			130 (81.3)	30 (18.8)		
Low	9 (7.8)	106 (92.2)			13 (15.9)	69 (84.1)			88 (73.9)	31 (26.1)		
Country of birth			.41	.07			.92	.02			.64	.04
Australia	7 (3.2)	214 (96.8)			24 (14.5)	141 (85.5)			176 (78.6)	48 (21.4)		
Elsewhere	5 (6.1)	77 (93.9)			8 (12.9)	54 (87.1)			64 (75.3)	21 (24.7)		
Smoking			**<.001**	**.28**			.30	.11			.23	.09
Current smoker	3 (37.5)	5 (62.5)			2 (40.0)	3 (60.0)			5 (55.6)	4 (44.4)		
Non-smoker	9 (3.1)	286 (96.9)			30 (13.5)	192 (86.5)			235 (78.3)	65 (21.7)		
Parity			.67	.04			.53	.05			.22	.08
Multiparous	6 (3.3)	176 (96.7)			21 (15.7)	113 (84.3)			138 (75.0)	46 (25.0)		
Primiparous	6 (5.0)	115 (95.0)			11 (11.8)	82 (88.2)			102 (81.6)	23 (18.4)		
Past mental health			**<.001**	**.31**			**.002**	**.23**			1.0	–
No	4 (1.5)	256 (98.5)			21 (10.8)	173 (89.2)			206 (77.7)	59 (22.3)		
Yes	8 (18.6)	35 (81.4)			11 (33.3)	22 (66.7)			34 (77.3)	10 (22.7)		
Body mass index			.40	.07			.33	.08			.46	.04
Non-obese	7 (3.2)	211 (96.8)			27 (16.7)	135 (83.3)			173 (77.9)	49 (22.1)		
Obese	4 (6.7)	56 (93.3)			5 (9.8)	46 (90.2)			51 (82.3)	11 (17.7)		

Note: frequency and percentage for women who responded

^±^At least probable minor antepartum depression = EPDS ≥ 13/Non-depressed = EPDS < 13; At least probable minor postpartum depression = EPDS ≥ 10/ Non-depressed EPDS < 10 postpartum

* Non-responders at 26-weeks postpartum includes women lost to follow up (9 women lost during pregnancy and 15 women lost postpartum)

^#^*p* = Continuity correction reported for 2 × 2 table

Phi = effect size and interpreted using Cohen’s criteria: .10 = small effect; .30 = medium effect; .50 = large effect

Statistically significant values are shown in **bold**

### Internal consistency reliability

The internal consistency reliability of the EPDS was: α = .85 during pregnancy at both time-points (baseline and 36-weeks), and α = .89 and .86 following birth (6- and 26-weeks). The PHQ-2 demonstrated high mean inter-item correlations (MIC) both during pregnancy (MIC = .55 and .59) and postpartum (MIC = .60, and .51).

### Incidence of EPDS and PHQ-2 positive screen tests

Table 2 presents the frequency and percentage (with 95% CI) of women with *probable major depression* and *at least probable minor depression* (EPDS) and positive screens (PHQ-2) at four time points over pregnancy (baseline, 36-weeks) and postpartum (6-, 26-weeks). The incidence of *probable major depression* was 2.3 and 1.8% during pregnancy (EPDS ≥ 15) and 5.6 and 6.2% postpartum (EPDS ≥ 13). The incidence of *at least probable minor depression* was 4.0 and 4.4% during pregnancy (EPDS ≥ 13) and 13.1 and 14.1% postpartum (EPDS ≥ 10). Positive screens on the PHQ-2 were highest using the alternative dichotomous (yes/no) method (incidence ranging 32.9–42.9%) and lowest using a cut-point of ≥ 3 (incidence ranged 4.4–7.5%) (Table 2).

**Table 2:** Frequency of women with depression^±^, and case-identification using two methods* on the PHQ-2

	Pregnancy		Postpartum	
	Baseline *n* = 303	36-weeks *n* = 275	6-weeks *n* = 252	26-weeks *n* = 227
	*n*	*n*	*n*	*n*
	*%*	*%*	*%*	*%*
	(95% CI)	(95% CI)	(95% CI)	(95% CI)
Probable major depression^±^	7	5	14	14
	2.3	1.8	5.6	6.2
	(0.9–4.7)	(0.6–4.2)	(3.1–9.2)	(3.4–10.1)
At least probable minor depression^±^	12 4.0	12 4.4	33 13.1	32 14.1
	(2.1–6.8)	(2.3–7.5)	(9.2–17.9)	(9.9–19.3)
PHQ-2 total score ≥ 2	49	58	50	35
	16.2	21.1	19.8	15.4
	(12.2–20.8)	(16.4–26.4)	(15.1–25.3)	(11.0–20.8)
PHQ-2 total score ≥ 3	14	20	19	10
	4.6	7.3	7.5	4.4
	(2.6–7.6)	(4.5–11.0)	(4.6–11.5)	(2.1–8.0)
PHQ-2 Screen positive	103	118	83	77
	33.9	42.9	32.9	33.9
	(28.7–39.6)	(37.0–49.0)	(27.2–39.1)	(27.8–40.5)
Anhedonia^a^ (no depressed mood)	80	96	61	52
	26.4	34.9	24.2	22.9
	(21.5–31.8)	(29.3–40.9)	(19.1–30.0)	(17.6–28.9)
Depressed mood^b^ (no anhedonia)	68	71	68	57
	22.4	25.8	27.0	25.1
	(17.9–27.6)	(20.8–31.4)	(21.6–32.9)	(19.6–31.3)
Depressed mood and anhedonia^c^	45	49	46	32
	14.9	17.8	18.3	14.1
	(11.0–19.4)	(13.5–22.9)	(13.7–23.6)	(9.9–19.3)

95% CI: 95% confidence interval for a sample proportion with binomial (Clopper Pearson) exact

^±^Probable major depression: EPDS cut-off score ≥ 15 during pregnancy and ≥ 13 postpartum; At least probable minor depression: EPDS cut-off score ≥ 13 in pregnancy and ≥ 10 postpartum

*PHQ-2: Total score method with two cut-points (≥ 2 and ≥ 3); Categorical method: Screen positive to either Question 1 OR Question 2

**PHQ-2 Question completion: Categories of positive responses on PHQ-2 questions

^a^Positive response to PHQ-2 Question 1 and negative response on question 2

^b^Positive response to PHQ-2 Question 2 and negative response to question 1

^c^Positive response to PHQ-2 Question 1 and question 2

**Fig. 2:**
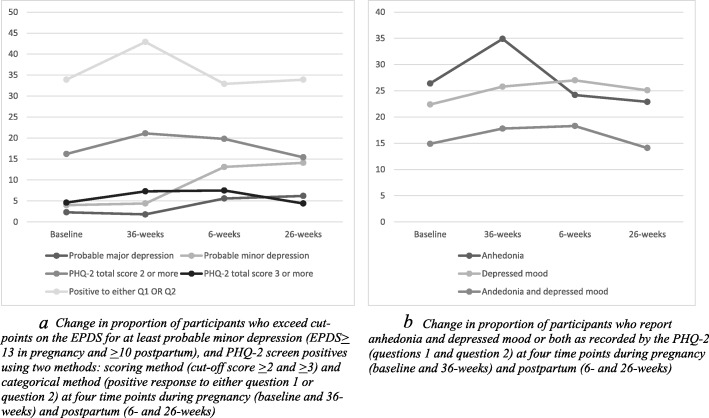
**a** Change in proportion of participants who exceed cut-points on the EPDS for at least probable minor depression (EPDS≥ 13 in pregnancy and ≥ 10 postpartum), and PHQ-2 screen positives using two methods: scoring method (cut-off score ≥ 2 and ≥ 3) and categorical method (positive response to either question 1 or question 2) at four time points during pregnancy (baseline and 36-weeks) and postpartum (6- and 26-weeks). Fig. 2**b** Change in proportion of participants who report anhedonia and depressed mood or both as recorded by the PHQ-2 (questions 1 and question 2) at four time points during pregnancy (baseline and 36-weeks) and postpartum (6- and 26-weeks)

Figure 2 presents data in a visual format. Fig. 2a shows probable minor depression (EPDS) was relatively stable during pregnancy and increased following birth (6-weeks). Cochran’s Q Test revealed the difference was significant (*n* = 207, *Q* = 34.82, *df* 3, *p* < .001). Pairwise comparisons confirmed the difference was significant from 36-weeks of pregnancy to 6-weeks postpartum (*p* < .001). Similar results were seen for major depression (*n* = 207, *Q* = 11.05, *df* 3, *p =* .01). Pairwise comparisons showed the difference was again seen between 36-weeks of pregnancy and 6-weeks postpartum (*p* = .01). No significant differences were observed for change in proportions of participants who screened positive for PHQ-2, regardless of method.

### Mood and anhedonia during pregnancy and postpartum (PHQ-2)

Figure 2b shows the incidence of anhedonia (PHQ-2: positive Q1, negative Q2) increased during pregnancy before reducing sharply in the early weeks following birth. Cochran’s Q Test revealed the difference was significant (*n* = 207, *Q* = 14.52, *df* 3, *p* = .002). Pairwise comparisons confirmed the difference was significant between booking and 36-weeks of pregnancy (*p* = .01), and between 36-weeks of pregnancy and 6-weeks postpartum (*p* = .01). No significant difference was seen for depressed mood (positive Q2, negative Q1), or for depressed mood *and* anhedonia (positive Q1 *and* Q2).

### Screening accuracy: ROC analyses

Table 3 presents findings of the ROC analysis for the PHQ-2 to detect *probable major depression* during pregnancy (EPDS ≥ 15) and postpartum (EPDS ≥ 13) and *at least probable minor depression* during pregnancy (EPDS ≥ 13) and postpartum (EPDS ≥ 10). At the ICHOM recommended cut-point of ≥ 3 for major depression the AUC was fair during pregnancy (AUC = .77), good in early postpartum (AUC = .88) and poor in late postpartum (AUC = .67). Reducing the cut-point to ≥ 2 improved the diagnostic accuracy (AUC = .88–.93) as did the dichotomous method (AUC = .79–.86). For minor depression, the PHQ-2 cut-off ≥ 3 was poor to fair (AUC = .58–.74). Reducing the cut-point to ≥ 2 improved diagnostic accuracy (AUC = .76–.94) as did the alternative dichotomous method (yes/no) (AUC = .78–.84).

**Table 3:** ROC Analysis of the PHQ-2 (cut-points ≥2, ≥ 3, yes/no) with EPDS* as criterion standard

Test Variable	AUC	Standard Error	Significance (*p*)	Lower bound	Upper bound
**PHQ-2** ≥ **3**					
*Probable major depression*					
Baseline	.77	.12	.02	.54	1.0
36-weeks	.77	.13	.04	.51	1.0
6-weeks postpartum	.88	.07	<.001	.75	1.0
26-weeks postpartum	.67	.09	.04	.49	.82
*At least probable minor depression*					
Baseline	.74	.09	.006	.55	.92
36-weeks	.72	.09	.009	.54	.90
6-weeks postpartum	.68	.06	.001	.57	.80
26-weeks postpartum	.58	.06	.13	.47	.70
**PHQ-2** ≥ **2**					
*Probable major depression*					
Baseline	.93	.02	<.001	.89	.97
36-weeks	.90	.03	.002	.84	.96
6-weeks postpartum	.92	.02	<.001	.89	.96
26-weeks postpartum	.88	.06	<.001	.77	.98
*At least probable minor depression*					
Baseline	.94	.02	<.001	.91	.97
36-weeks	.91	.02	<.001	.87	.95
6-weeks postpartum	.80	.05	<.001	.71	.90
26-weeks postpartum	.76	.05	<.001	.65	.86
**PHQ-2 Positive Yes/No**					
*Probable major depression*					
Baseline	.84	.04	.002	.76	.92
36-weeks	.79	.06	.026	.68	.90
6-weeks postpartum	.86	.03	<.001	.80	.91
26-weeks postpartum	.81	.05	<.001	.72	.91
*At least probable minor depression*					
Baseline	.84	.03	<.001	.78	.91
36-weeks	.80	.04	<.001	.72	.87
6-weeks postpartum	.78	.04	<.001	.70	.87
26-weeks postpartum	.78	.04	<.001	.69	.86

The EPDS was used as the reference standard

*AUC* Area under the curve; Interpreted as AUC = .60–.70 = poor, .70–.80 = fair, .80–0.90 = good, .90–1.0 = excellent

A*t least probable minor depression in pregnancy:* pregnancy = ≥ 13 and postpartum = ≥ 10

*Probable major depression:* pregnancy = ≥ 15 and postpartum = ≥ 13

### Antepartum probable major depression: PHQ-2 screening accuracy

Table 4 presents the screening accuracy of the PHQ-2 at 4 time-points for *probable major depression*. During pregnancy (booking, 36-weeks) the ICHOM recommended PHQ-2 cut-point of ≥ 3, correctly classified 57 and 60% of women with probable major depression but consequently missed 43 and 40% of at-risk women. Some 3.4 and 6.3% of women with no probable major depression would have been asked to complete the EPDS. A lowered PHQ-2 cut-point correctly classified 100% of women with probable major depression at both booking and 36-weeks. However, 14.2 and 19.6% of women under the threshold for probable major depression would have been asked to complete the EPDS. Using the alternative categorical approach (positive response to either Q1 or Q2), though 100% of women with probable major depression were correctly identified, the EPDS would have been administered to 32.4 and 41.9% of women under the threshold. Among those who screened positive on the PHQ-2, the probability of having probable major antepartum depression (EPDS ≥ 15) at booking was greatest using a cut-point ≥ 3 and lowest using the alternative method (PPV = 29% vs 7%, respectively). Similar results were seen at 36-weeks (PPV = 15% vs 4%) (Table 4).

**Table 4:** Screening accuracy of the PHQ-2* during pregnancy and postpartum using the EPDS as reference standard for *probable major depression*

	Antepartum					
*n* % (95% CI)	Baseline *n* = 303			36-weeks *n* = 275		
	Positive	≥ 2	≥ 3	Positive	≥ 2	≥ 3
True positive	7	7	4	5	5	3
False negative	0	0	3	0	0	2
True negative	200	254	286	157	217	253
False positive	96	42	10	113	53	17
Sensitivity	1.0 (0.65–1.0)	1.0 (0.65–1.0)	0.57 (0.25–0.84)	1.0 (0.57–1.0)	1.0 (0.57–1.0)	0.60 (0.23–0.88)
Specificity	0.68 (0.62–0.73)	0.86 (0.81–0.89)	0.97 (0.94–0.98)	0.58 (0.52–0.64)	0.80 (0.75–0.85)	0.94 (0.90–0.96)
PPV	0.07 (0.03–0.13)	0.14 (0.07–0.27)	0.29 (0.12–0.55)	0.04 (0.02–0.10)	0.09 (0.04–0.19)	0.15 (0.05–0.36)
NPV	1.0 (0.98–1.0)	1.0 (0.99–1.0)	0.99 (0.97–1.0)	1.0 (0.98–1.0)	1.0 (0.98–1.0)	0.99 (0.97–1.00)
+LR	3.09 (2.62–3.63)	7.04 (5.33–9.33)	16.79 (6.98–40.97)	2.39 (2.08–2.75)	5.10 (4.00–6.49)	9.52 (4.07–22.31)
-LR	0 -	0 -	0.44 (0.19–1.04)	0 -	0 -	0.43 (0.15–1.25)
**Postpartum**						
	6-weeks *n* = 252			26-weeks *n* = 227		
	Positive	≥ 2	≥ 3	Positive	≥ 2	≥ 3
True positive	14	14	11	13	12	5
False negative	0	0	3	1	2	9
True negative	169	202	230	149	190	208
False positive	69	36	8	64	23	5
Sensitivity	1.00 (0.79–1.00)	1.00 (0.79–1.00)	0.79 (0.52–0.92)	0.93 (0.69–0.99)	0.86 (0.60–0.96)	0.36 (0.16–0.61)
Specificity	0.71 (0.65–0.76)	0.85 (0.80–0.89)	0.97 (0.94–0.98)	0.70 (0.64–0.76)	0.89 (0.84–0.93)	0.98 (0.95–0.99)
PPV	0.17 (0.10–0.26)	0.28 (0.18–0.42)	0.58 (0.36–0.77)	0.17 (0.10–0.27)	0.34 (0.21–0.51)	0.50 (0.24–0.76)
NPV	1.00 (0.98–1.00)	1.00 (0.98–1.0)	0.99 (0.96–1.0)	0.99 (0.96–1.00)	0.99 (0.96–1.00)	0.96 (0.92–0.98)
+LR	3.45 (2.83–4.21)	6.62 (4.89–8.93)	23.12 (11.22–48.70)	3.10 (2.40–3.97)	7.94 (5.11–12.34)	15.52 (4.99–46.42)
-LR	0 -	0 -	0.22 (0.08–0.61)	0.10 (0.02–0.68)	0.16 (0.04–0.58)	0.66 (0.45–0.97)

*Scoring method at cut-point ≥ 2 and ≥ 3 and categorical method (positive response to either or both questions)

### Postpartum probable major depression: PHQ-2 screening accuracy

Following birth (6-weeks, 26-weeks), the PHQ-2 cut-point of ≥ 3 correctly classified 79 and 36% of women with probable major depression but missed 21 and 64% high-risk women. Only 3.4 and 2.3% of women under the threshold for probable major depression would have been asked to complete the EPDS. At a lowered cut-point of ≥ 2, 100% of women with probable major depression were correctly classified at baseline. While 86% of women with probable depression were correctly classified at 26-weeks, 14% of high-risk women were missed. At the lowered cut-point 15.1 and 10.8% of women with no probable major depression would have been asked to complete the EPDS. While the alternative method achieved the highest overall sensitivity following birth, correctly classifying 100% of women at 6-weeks and 93% of women at 26-weeks, some 29 and 30% of women with no probable depression would have been asked to complete the EPDS. Among those who screened positive on the PHQ-2, the probability of having major postpartum depression (EPDS ≥ 13) at 6-weeks was greatest using a cut-point ≥ 3 and lowest using the alternative categorical method (PPV = 58% vs 17%, respectively). Similar results were seen at 26-weeks (PPV = 50% vs 17%) (Table 4).

### Antepartum probable minor depression: PHQ-2 screening accuracy

Table 5 presents the screening accuracy of the PHQ-2 at 4 time-points for *at least probable minor depression*. During pregnancy (booking, 36-weeks) a PHQ-2 cut-point of ≥ 3, correctly classified 50% of women with probable minor depression at both time-points but 50% of at-risk women were missed. Some 2.7 and 5.3% of low-risk women would have been asked to complete the EPDS unnecessarily. A lowered PHQ-2 cut-point correctly classified 100% of women with probable minor depression at both booking and 36-weeks. However, 12.7 and 17.5% low-risk women would have been asked to complete the EPDS. Using the alternative categorical approach (positive response to either Q1 or Q2), though 100% of women with probable minor depression were correctly identified, the EPDS would have been administered to 31.2 and 40.3% of women below the EPDS threshold for probable minor depression. Among those who screened positive on the PHQ-2, the probability of also screening positive on the EPDS for at least probable minor antepartum depression (EPDS ≥ 13) at booking was greatest using a cut-point ≥ 3 and lowest using the alternative method (PPV = 43% vs 12%, respectively). Similar results were seen at 36-weeks (PPV = 30% vs 10%) (Table 5).

**Table 5:** Screening accuracy of the PHQ-2* during pregnancy and postpartum using the EPDS as reference standard for *at least probable minor depression*

	**Antepartum**					
*n* % (95% CI)	Baseline *n* = 303			36-weeks *n* = 275		
	Positive	≥ 2	≥ 3	Positive	≥ 2	≥ 3
True positive	12	12	6	12	12	6
False negative	0	0	6	0	0	6
True negative	200	254	283	157	217	249
False positive	91	37	8	106	46	14
Sensitivity	1.0 (0.76–1.0)	1.0 (0.76–1.0)	0.50 (0.25–0.75)	1.0 (0.76–1.0)	1.0 (0.76–1.0)	0.50 (0.25–0.75)
Specificity	0.69 (0.63–0.74)	0.87 (0.83–0.91)	0.97 (0.95–0.99)	0.60 (0.54–0.65)	0.83 (0.78–0.87)	0.95 (0.91–0.97)
PPV	0.12 (0.07–0.19)	0.25 (0.15–0.38)	0.43 (0.21–0.67)	0.10 (0.06–0.17)	0.21 (0.12–0.33)	0.30 (0.15–0.52)
NPV	1.0 (0.98–1.0)	1.0 (0.99–1.0)	0.98 (0.96–0.99)	1.0 (0.98–1.0)	1.0 (0.98–1.0)	0.98 (0.95–0.99)
+LR	3.20 (2.70–3.79)	7.87 (5.82–10.63)	18.52 (7.49–44.17)	2.48 (2.14–2.87)	5.71 (4.40–7.43)	9.4 (4.39–20.12)
-LR	0 -	0 -	0.51 (0.29–0.91)	0 -	0 -	0.53 (0.30–0.93)
**Postpartum**						
	6-weeks *n* = 252			26-weeks *n* = 227		
	Positive	≥ 2	≥ 3	Positive	≥ 2	≥ 3
True positive	27	24	13	26	19	6
False negative	6	9	20	6	13	26
True negative	163	193	213	144	179	191
False positive	56	26	6	51	16	4
Sensitivity	0.82 (0.66–0.91)	0.73 (0.56–0.85)	0.39 (0.25–0.56)	0.81 (0.65–0.91)	0.59 (0.42–0.75)	0.19 (0.09–0.35)
Specificity	0.74 (0.68–0.80)	0.88 (0.83–0.92)	0.97 (0.94–0.99)	0.74 (0.67–0.80)	0.92 (0.87–0.95)	0.98 (0.95–0.99)
PPV	0.33 (0.23–0.43)	0.48 (0.35–0.62)	0.68 (0.46–0.85)	0.34 (0.24–0.45)	0.54 (0.38–0.70)	0.60 (0.31–0.83)
NPV	0.96 (0.93–0.98)	0.96 (0.92–0.98)	0.91 (0.87–0.94)	0.96 (0.92–0.98)	0.93 (0.89–0.96)	0.88 (0.83–0.92)
+LR	3.20 (2.43–4.22)	6.11 (4.04–9.30)	14.59 (5.87–35.21)	3.10 (2.33–4.15)	7.24 (4.18–12.54)	8.95 (2.73–30.61)
-LR	0.25 (0.12–0.51)	0.31 (0.18–0.54)	0.62 (0.47–0.82)	0.25 (0.12–0.53)	0.44 (0.29–0.67)	0.83 (0.70–0.98)

*Scoring method at cut-point ≥ 2 and ≥ 3 and categorical method (positive response to either or both questions)

### Postpartum probable minor depression: PHQ-2 screening accuracy

Following birth (6-weeks, 26-weeks), the PHQ-2 cut-point of ≥ 3 correctly classified 39 and 19% of women with at least probable minor depression but missed 61 and 81% of at-risk women. Only 2.1 and 1.5% of women under the threshold for minor depression would have been asked to complete the EPDS. At a PHQ-2 lowered cut-point of ≥ 2, 73 and 59% of women with probable minor depression were correctly classified at baseline and 26-weeks respectively. Consequently, some 27 and 41% of at-risk women were missed. At the lowered cut-point, 8.9 and 6.1% of low-risk women would have been asked to complete the EPDS unnecessarily. While the alternative method achieved the highest overall sensitivity following birth, correctly classifying 82% of women at 6-weeks and 81% of women at 26-weeks, 19% of low-risk women would have been asked to complete the EPDS at both postpartum time-points. Among those who screened positive on the PHQ-2, the probability of also screening positive for at least probable minor postpartum depression (EPDS ≥ 10) at 6-weeks was greatest using a cut-point ≥ 3 and lowest using the alternative categorical method (PPV = 68% vs 33%, respectively). Similar results were seen at 26-weeks (PPV = 60% vs 34%) (Table 5).

## Discussion

This study evaluated the screening accuracy of the PHQ-2 to inform the future use of the ICHOM core outcome set for pregnancy and childbirth in clinical practice and research. Two methods of case-identification were used: a scoring method with two cut-points (≥ 2 / ≥ 3) and an alternative dichotomous method. The EPDS was used as the reference standard at recommended cut-points [16, 34, 35] and demonstrated acceptable internal consistency reliability across all four time-points (a = .85–89). In contrast the mean inter-item correlations of the PHQ-2 (MIC = .51–.60) were high [42], suggesting some possible overlap between anhedonia and depressive symptoms during the perinatal period.

At the ICHOM recommended PHQ-2 cut-point of ≥ 3, screening accuracy to detect probable major depression was fair during pregnancy (AUC = .77), and poor to good postpartum period (AUC = .67–.88). While the recommended cut-point demonstrated low sensitivity during pregnancy (57–60%) and postpartum (36–79%) and missed an unacceptably high number of women with probable major depression, specificity remained high (94–98%) thus minimizing response burden, an identified priority to the ICHOM team [14]. Lowering the cut-point to ≥ 2 achieved the highest screening accuracy for probable major depression of the three methods, demonstrated by good to excellent AUC values at all timepoints (AUC = .88–.93) and high sensitivity (86–100%). Further, specificity was moderate throughout (80–89%). In contrast, while an alternative dichotomous method achieved only fair to good diagnostic accuracy (AUC .79–.86), it did demonstrate the highest overall sensitivity (100% during pregnancy and early postpartum and 93% late postpartum). Specificity was however lower than the other two methods (58–71%). The alternative method thus identified almost all at risk women but at the cost of increased response-burden.

For at least probable minor depression, screening accuracy was highest using the PHQ-2 cut-point of ≥ 2 (AUC = .76–.94) and lowest using the PHQ-2 cut-point of ≥ 3 (AUC = .58–.74). While sensitivity was lowest at a cut-point of ≥ 3 (19 to 50%) it did demonstrate the highest specificity (95–98%). In contrast, while the alternative dichotomous method had the highest sensitivity (81–100%), specificity was low (60–74%).

The ability to compare our findings was hindered by a lack of comparable perinatal studies. A systematic review which evaluated the accuracy of screening instruments for perinatal women [18], identified sparse evidence pertaining to the PHQ-2. Further, the reporting of two similar tools within the literature compounds the challenge of comparing findings as the Whooley Questions are often confused with, and referred to, as the PHQ-2 and vice versa [27, 28]. The two tools are also referred to, or combined, as case-finding or case-identification methods [29, 45]. As such we compared our findings to the literature pertaining to both the PHQ-2 and the Whooley Questions, where appropriate to do so.

### Screening accuracy of case-identifying methods

The literature identifies two almost identical case-identifying methods – the PHQ-2 and the Whooley Questions. In terms of the PHQ-2, authors of a systematic review revealed a pooled sensitivity of 0.76 and specificity of 0.87 at a cut-point of ≥ 3 in the general population [27]. Consistent with the current study, sensitivity improved at a cut-point of ≥ 2 (sensitivity = 0.91) and specificity decreased (70%). Findings of that review are however limited by the largely mixed-gender, and recruitment from primary/secondary settings and substantial heterogeneity between studies (I^2^ = 81.8%). Only one included study by Smith and colleagues [46] reported findings for perinatal women. Similarly, a meta-analysis of the diagnostic accuracy of the Whooley Questions to identify major depression included ten studies with community samples [28] including two in peripartum women [45, 47]. Consistent with the current study, Bosanquet [28] reported a pooled sensitivity of 95% (95% CI: 0.88–0.97), and pooled specificity of 65% (95% CI: 0.55–0.74) using the dichotomous response format. Maternity-specific evidence regarding diagnostic accuracy is sparse. With a focus on postpartum depression, though Mann and Gilbody [29] identified seven studies reporting either case-identification method (PHQ-2 and Whooley Questions), only one study met inclusion criteria using clinical diagnostic criteria. The included study by Gjerdingen et al., [47] reported diagnostic accuracy of both the PHQ-2 and Whooley Questions. As a screening tool for major postpartum depression, Gjerdingen reported the Whooley method to have 100% sensitivity, 62% specificity, 11% PPV and 100% NPV, comparing favorably to the findings of the current study for at least probable major depression at six weeks postpartum (sensitivity: 100%, specificity: 71%, PPV: 17%, NPV: 100%). Though Gjerdingen evaluated the PHQ-2, cut-points were not evaluated preventing further comparison. In terms of the Whooley Questions, recent work by Littlewood and colleagues [48] revealed the alternative method (positive response to either or both questions) to demonstrate acceptable sensitivity and specificity but low predictive value during pregnancy (20 weeks: sensitivity: 85%, specificity: 83.7%, PPV: 37.4) and postpartum (3–4 months: sensitivity: 85.7%, specificity: 80.6%, PPV: 31.4) periods which is consistent with current findings. Current study findings for at least probable minor depression at similar timepoints reveal similar results following birth, but greater sensitivity during pregnancy (baseline: sensitivity: 100%, specificity: 69%, PPV: 12) and postpartum (26 weeks: sensitivity: 81%, specificity: 74%, PPV: 34). The disparity in findings likely reflect the difference in reference standards used; the current study used the EPDS, while Littlewood used the Client Interview Schedule- Revised [49]. Further, the impact of question wording differences in terms of time format (last-2 weeks versus past month) is not yet known.

An important observation seen in the current study but not previously evaluated by others is the significant increase in anhedonia seen during pregnancy and significant decrease following birth which was not seen with the PHQ-2 depression question. It is possible that women may experience low mood as pregnancy progresses without a significant change in levels of depressive symptoms. The impact of anhedonia may then artificially inflate the number of women meeting the screening threshold. Further research to identify the impact of each item during the perinatal period is warranted.

### EPDS as a reference standard

Consistent with the current study, the PHQ-2 has been evaluated as a modified dichotomous yes/no screening tool using the EPDS as the reference standard (37, 38). Using an EPDS cut-off score of ≥ 13 to denote *probable major depression* during pregnancy (15-weeks, 30-weeks) and postpartum (6–16 weeks), Bennett and colleagues [37] reported the ‘modified version of the PHQ-2’ to have high sensitivity (80–93%) and specificity (75–86%), with sensitivity being highest during pregnancy and lower postpartum. At the same EPDS cut-point (≥ 13) the current study showed a slightly higher sensitivity (93–100%) and lower specificity (60–71%). These differences may be attributed to sample characteristics and research methodology. Bennett’s cross-sectional study recruited young, low income, less educated women with higher rates of depressive symptoms compared to the current study. Of significance, Bennett reported evaluating a modified version of the PHQ-2 which were the Whooley Questions (*During the past month …*), further demonstrating the inconsistency and potential confusion in instrument reporting.

Consistent with the current study, Chae et al., [38] evaluated the PHQ-2 using the dichotomous method against the EPDS as the reference standard using a cut-point ≥ 13 in a sample of women (*n* = 200) attending well-child clinics in the USA at 6 weeks and 6 months postpartum. Chae reported 100% sensitivity and 79% specificity, which compares well to the 93–100% sensitivity and 70–71% specificity in the current study. More recently, Howard et al., [50] compared the Whooley Questions and the EPDS (cut-point ≥ 13) to clinical diagnostic criteria in a cross-sectional study with 545 women attending their first booking appointment. These authors reported low sensitivity for the Whooley Questions (41%) and EPDS (59%), with a concomitant high specificity (94–95%). While few comparable studies exist for the Whooley Questions, sensitivity of the EPDS is lower than usually reported and might be explained by the large sample size, diverse sample of women and delays in administering the diagnostic interview.

### Clinical implications

The ICHOM recommended cut-point of ≥ 3 on the PHQ-2 to screen for probable *major depression* is consistent with recommendations by the scale developer [15] despite being based primarily on a validation study with patients in primary and secondary care. Our findings demonstrate that at this cut-point an unacceptably high number of ‘*at-risk’* women were missed and would not have received the follow-up EPDS. While a cut-point of ≥ 2 provided highest accuracy in terms of area under the curve, and optimal sensitivity and specificity during pregnancy, screening accuracy was lower following birth. Further, in clinical practice where the identification of high-risk women is crucial, we would argue that an alternative dichotomous method missed the least number of at-risk women and is the most appropriate method of case-identification during the perinatal period. Further, this method maintained the highest sensitivity even with a lowered EPDS cut-point to denote *at least probable minor depression*. This finding indicates that the alternative dichotomous method would better identify women with lower levels of depressive symptoms compared to the scoring method to better inform clinical-decision making. While a higher false positive rate is noted with this method, we argue that where universal screening using the EPDS is already currently practiced, this method could positively impact response burden for many women.

In Australia government-funded projects are working towards a standardized approach to outcome reporting in maternity-related practice and research to improve data-synthesis and outcomes for women and their babies. Our findings offer important evidence and recommendations to support standardization.

### Strengths and limitations

Despite our best efforts, our findings do have some limitations. Our study included a cohort of women from one Australian birthing facility and identified a low prevalence of women with probable major depression during pregnancy (around 2%) and postpartum (around 6%) which contributes to a less precise measure. While our prevalence rates are slightly less than those reported by Gaynes et al., [51] during pregnancy (3.1–4.9%), our findings are comparable to postpartum rates (1.0–5.9%). Recruiting larger, more diverse samples would improve precision of the prevalence estimate and generalizability of findings. We used the EPDS as the reference standard rather than a clinical diagnosis of depression which would generally be considered gold standard. However, the aim here was to evaluate the PHQ-2 against the EPDS as would occur in real-life clinical practice using the ICHOM core outcome set and not to diagnose depression. Our findings were however strengthened by the comprehensive nature of our analysis which included using two case-identification methods over four time-points. Further, we applied widely-accepted EPDS cut-points to denote both probable major depression and at least probable minor depression.

### Conclusions

ICHOM recommend the PHQ-2 to screen women using a scoring method at a defined cut-point. The recommended method has been shown to be inadequate as a probable perinatal depression case-identification method using the EPDS as the reference standard. To optimise the number of women identified as ‘*at-risk*’ in clinical practice, we recommend a dichotomous two-item case-identification method which is consistent with recommendations of international guidelines [7] and has been shown to be acceptable to women [48]. Further, to address the current confusion surrounding the use and reporting of the case-identification method [27, 28], we recommend the use of the Whooley Questions rather than the PHQ-2. The use and reporting of consistent question wording and response format will improve future outcome reporting and synthesis.

## Supplementary information

**Additional file 1.** STARD Checklist.

**Additional file 2.** Edinburgh Postnatal Depression Scale: Questions and screening accuracy.

## Data Availability

The de-identified dataset used and analysed for this study is available from the corresponding author upon reasonable request so that appropriate data transfer agreements can be established.

## Abbreviations

AUC: Area under the curve
CI: Confidence interval
EPDS: Edinburgh postnatal depression scale
ICHOM: International consortium for health outcomes measurement
MIC: Mean inter-item correlation
NLR: Negative likelihood ratio
NPV: Negative predictive value
PHQ: Patient health questionnaire
PLR: Positive likelihood ration
PPV: Positive predictive value
ROC: Receiver operating characteristic
UK: United Kingdom
