# Supplementary material for: Comparison of screening accuracy of the Patient Health Questionnaire-2 using two case-identification methods during pregnancy and postpartum
Source: BMC Pregnancy Childbirth. 2020 Apr 14;20:211. doi: 10.1186/s12884-020-02891-2 (PMC7158032; doi:10.1186/s12884-020-02891-2)
Supplement: Supplementary file 1 — Additional file 1. STARD Checklist. [file 12884_2020_2891_MOESM1_ESM.docx]

Additional File 1

STARD Checklist.

|  | **Section & Topic** | **No** | **Item** | **Reported on page #** |
| --- | --- | --- | --- | --- |
|  |  |  |  |  |
|  | **TITLE OR ABSTRACT** |  |  |  |
|  |  | **1** | Identification as a study of diagnostic accuracy using at least one measure of accuracy  (such as sensitivity, specificity, predictive values, or AUC) | In Abstract: Methods. ROC |
|  | **ABSTRACT** |  |  |  |
|  |  | **2** | Structured summary of study design, methods, results, and conclusions  (for specific guidance, see STARD for Abstracts) | See STARD for ABSTRACT CHECKLIST |
|  | **INTRODUCTION** |  |  |  |
|  |  | **3** | Scientific and clinical background, including the intended use and clinical role of the index test | 🗸 Page 3-4 |
|  |  | **4** | Study objectives and hypotheses | P4 Line 11 |
|  | **METHODS** |  |  |  |
|  | *Study design* | **5** | Whether data collection was planned before the index test and reference standard  were performed (prospective study) or after (retrospective study) | P4 Line 17 |
|  | *Participants* | **6** | Eligibility criteria | P5 Section 2.2 |
|  |  | **7** | On what basis potentially eligible participants were identified  (such as symptoms, results from previous tests, inclusion in registry) | P6 Line 11 Consecutive |
|  |  | **8** | Where and when potentially eligible participants were identified (setting, location and dates) | Section 2.4 procedures |
|  |  | **9** | Whether participants formed a consecutive, random or convenience series | P6 Line 11 |
|  | *Test methods* | **10a** | Index test, in sufficient detail to allow replication | Page 6 lines 20-22 |
|  |  | **10b** | Reference standard, in sufficient detail to allow replication | Page 6 Lines 22 – Page 7 Line 2 |
|  |  | **11** | Rationale for choosing the reference standard (if alternatives exist) | P2 Line 17  P18 Line 17 |
|  |  | **12a** | Definition of and rationale for test positivity cut-offs or result categories  of the index test, distinguishing pre-specified from exploratory | ICHOM recommendation Page 1 |
|  |  | **12b** | Definition of and rationale for test positivity cut-offs or result categories  of the reference standard, distinguishing pre-specified from exploratory | Page 6 Line 1 |
|  |  | **13a** | Whether clinical information and reference standard results were available  to the performers/readers of the index test | NA conducted at analysis stage only |
|  |  | **13b** | Whether clinical information and index test results were available  to the assessors of the reference standard | NA Conducted at analysis stage only |
|  | *Analysis* | **14** | Methods for estimating or comparing measures of diagnostic accuracy | P7 Line 22 |
|  |  | **15** | How indeterminate index test or reference standard results were handled | NA |
|  |  | **16** | How missing data on the index test and reference standard were handled | P7 Line 7 |
|  |  | **17** | Any analyses of variability in diagnostic accuracy, distinguishing pre-specified from exploratory | NA |
|  |  | **18** | Intended sample size and how it was determined | P5 Line 9 |
|  | **RESULTS** |  |  |  |
|  | *Participants* | **19** | Flow of participants, using a diagram | STARD Flow chard Supp Figure 1 |
|  |  | **20** | Baseline demographic and clinical characteristics of participants | Page 8 Line 10  Table 1 |
|  |  | **21a** | Distribution of severity of disease in those with the target condition | P9 Section 3.3  Table 2 |
|  |  | **21b** | Distribution of alternative diagnoses in those without the target condition | NA |
|  |  | **22** | Time interval and any clinical interventions between index test and reference standard | 4 time points clearly described throughout |
|  | *Test results* | **23** | Cross tabulation of the index test results (or their distribution)  by the results of the reference standard | SUPP Figure 1 STARD diagram |
|  |  | **24** | Estimates of diagnostic accuracy and their precision (such as 95% confidence intervals) | P10 Section 3.5  Table 3 |
|  |  | **25** | Any adverse events from performing the index test or the reference standard | NA |
|  | **DISCUSSION** |  |  |  |
|  |  | **26** | Study limitations, including sources of potential bias, statistical uncertainty, and generalisability | P18 Section 4.4 |
|  |  | **27** | Implications for practice, including the intended use and clinical role of the index test |  |
|  | **OTHER INFORMATION** |  |  |  |
|  |  | **28** | Registration number and name of registry | P17 Section 4.3 |
|  |  | **29** | Where the full study protocol can be accessed | No study protocol |
|  |  | **30** | Sources of funding and other support; role of funders | P20 Line 9 |
|  |  |  |  |  |

**STARD for Abstracts:**

| Section | | Item | Page |
| --- | --- | --- | --- |
|  | Identification as a study of diagnostic accuracy using at least one measure of accuracy (such as sensitivity, specificity, predictive values, or AUC) | 🗸 |  |
| **Background and Objectives** | Study objectives | 🗸 |  |
| **Methods** | Data collection: whether this was a prospective or retrospective study | 🗸 |  |
|  | Eligibility criteria for participants and settings where the data were collected | Partially described due to wordage |  |
|  | Whether participants formed a consecutive, random, or convenience series | 🗸  Consecutive |  |
|  | Description of the index test and reference standard | 🗸 |  |
| **Results** | Number of participants with and without the target condition included in the analysis | No due to word count |  |
|  | Estimates of diagnostic accuracy and their precision (such as 95% confidence intervals) | 🗸 |  |
| **Discussion** | General interpretation of the results | 🗸 |  |
|  | Implications for practice, including the intended use of the index test | 🗸 |  |
| **Registration** | Registration number and name of registry | NA |  |
