# Supplementary material for: Comparison of screening accuracy of the Patient Health Questionnaire-2 using two case-identification methods during pregnancy and postpartum
Source: BMC Pregnancy Childbirth. 2020 Apr 14;20:211. doi: 10.1186/s12884-020-02891-2 (PMC7158032; doi:10.1186/s12884-020-02891-2)
Supplement: Supplementary file 2 — Additional file 2. Edinburgh Postnatal Depression Scale: Questions and screening accuracy. [file 12884_2020_2891_MOESM2_ESM.docx]

**Additional File 2**

Edinburgh Postnatal Depression Scale: Questions and screening accuracy.

| Questions | Screening accuracy* |
| --- | --- |
| *Stem: In the past 7 days…*   1. *I have been able to see the funny side of things* 2. *I have looked forward with enjoyment to things* 3. *I have blamed myself unnecessarily when things went wrong* 4. *I have been anxious or worried for no good reason* 5. *I have felt scared or panicky for no very good reason* 6. *Things have been getting on top of me* 7. *I have been so unhappy that I have had difficulty sleeping* 8. *I have felt sad or miserable* 9. *I have been so unhappy that I have been crying* 10. *The thought of harming myself has occurred to me* | *Pregnancy: Mixed depression (Minor and major)*  Cut-point >10  pooled sensitivity 0.74 (95% CI: 0.65 – 0.82)  pooled specificity 0.86 (95% CI: 0.83 – 0.89)  Cut-point >13  pooled sensitivity 0.61 (95% CI: 0.5 – 0.72)  pooled specificity 0.94 (95% CI: 0.92 – 0.96)  Cut-point >15  pooled sensitivity 0.47 (95% CI: 0.35 – 0.60)  pooled specificity 0.98 (95% CI: 0.97 – 0.99)  *Pregnancy: Major depression*  Cut-point >10  pooled sensitivity 0.88 (95% CI: 0.89 – 0.94)  pooled specificity 0.88 (95% CI: 0.86 – 0.90)  Cut-point >13  Pooled sensitivity 0.83 (95% CI: 0.76 – 0.88) Pooled specificity 0.90 (95% CI: 0.88 – 0.92)  Cut-point >15  pooled sensitivity 0.72 (95% CI: 0.58 – 0.84)  pooled specificity 0.97 (95% CI: 0.95 – 0.98)  *Postnatal: Mixed depression (minor and major)*  Cut-point >10  pooled sensitivity 0.83 (95% CI: 0.81 – 0.86)  pooled specificity 0.85 (95% CI: 0.84 – 0.86)  Cut-point >13  Pooled sensitivity 0.68 (95% CI: 0.66 – 0.71)  Pooled specificity 0.92 (95% CI: 0.92 – 0.93)  Postnatal: *major depression*  Cut-point >10  Pooled sensitivity 0.95 (95% CI: 0.92 – 0.97) Pooled specificity 0.82 (95% CI: 0.80 – 0.84)  *Postnatal: major depression*  Cut-point >13  Pooled sensitivity 0.80 (95% CI: 0.77 – 0.83) Pooled specificity 0.93 (95% CI: 0.92 – 0.94 |

*Screening accuracy (National Collaborating Centre for Mental Health, 2018)

National Collaborating Centre for Mental Health. (2018). Antenatal and postnatal mental health. The Nice Guideline on clinical management and service guidance. Updated edition. National Clinical Guideline 192. Retrieved from <https://www.nice.org.uk/guidance/cg192/evidence/full-guideline-pdf-4840896925>
